# The Role of Nationality in Childhood Caries in Qatar

**DOI:** 10.1111/cdoe.13010

**Published:** 2024-10-30

**Authors:** Andrew John Spencer, Asmaa Othman AlKhtib, Mohamed Sultan Al Darwish, Hasaan Gassim Saad Mohame, Tintu Mathew, Ghanim Ali Al Mannai, Mohammed Al Thani, Mariam Abdulmalik, Johann de Vries, Loc Giang Do, Sergio Chrisopoulos

**Affiliations:** ^1^ Australian Research Centre for Population Oral Health The University of Adelaide Adelaide Australia; ^2^ Ministry of Public Health Doha Qatar; ^3^ College of Dental Medicine Qatar University Doha Qatar; ^4^ Hamad Medical Corporation (HMC) Doha Qatar; ^5^ Primary Health Care Corporation (PHCC) Doha Qatar; ^6^ School of Dentistry University of Queensland Brisbane Australia

**Keywords:** caries, child, primary dentition, Qatar, socio‐demographics

## Abstract

Childhood primary dentition caries prevalence and experience is higher among Qatari than non‐Qatari nationality children in Qatar but only this bivariate association has been described.

**Objectives:**

There were two objectives. First, to understand the variation of primary dentition caries among 4‐ to 8‐year‐old children in Qatar across nationality classified into four groups, and second, to explore whether the association persisted in the presence of socio‐demographic and behavioural indicators.

**Methods:**

The study used data from the Qatar Child Oral Health Survey 2017 (QCOHS 2017). Detailed information was collected through a parental dual‐language questionnaire and an oral epidemiological examination conducted by calibrated dentist examiners. Children in 20 kindergartens and 40 schools across Qatar were recruited. Data were weighted to represent the Qatar child population.

**Results:**

Overall, 1154 children aged 4–8 years old (48.9% female, 51.1% male) participated. Qatari children made up 26.3%, Non‐Qatari (N‐Q) Arabic children 44.2% N‐Q Indian sub‐continent 16.4% and N‐Q Other 13.1%. There were no significant differences by nationality for age or sex, but differences existed for kindergarten/school type and parents' highest level of education. Among behavioural indicators, Qatari and N‐Q Arabic children began toothbrushing later, and more N‐Q Other children brushed 2+ times a day and had made a check‐up visit in the last 12 months. More Qatari children were in the highest tertial for sugar intake and drank bottled water with no fluoride. All N‐Q children had a significantly lower prevalence and experience of caries. The means ratio (95% CI) for N‐Q Arabic (0.78; 0.65–0.94), Indian (0.58; 0.46–0.72) and other children (0.61; 0.42–0.88) were all significant against Qatari nationality children. Multivariable models showed an attenuation of the association with caries with the means ratio for N‐Q Arabic (0.92; 0.73–1.16), Indian (0.79; 0.57–1.11) and other children (0.94; 0.61–1.44) being non‐significant compared to Qatari nationality children. The variables which were significantly associated with caries were parental education, toothbrushing frequency, sugar intake and check‐up visiting in the last 12 months in the multivariable models.

**Conclusions:**

Primary dentition caries in children resident in Qatar differed by nationality. The association of primary dentition caries with nationality was markedly attenuated and non‐significant in the presence of socio‐demographic and behavioural variables, pointing towards the importance of these variables as the pathways to improving primary dentition caries prevalence and experience of children in Qatar.

## 1 Introduction

Nationality defines an ethnic collective with common origins and a sense of identity and shared culture. Having a common origin within an ethnic collective establishes social ties, implies shared social norms that may shape thought and behaviours and places people within a larger social system [1]. It has meaning in countries with a strong native or indigenous occupancy and large numbers of recent immigrant or guest residents. This is the situation in Qatar.

Qatar has a small native population and large numbers of non‐Qataris who are either guests or short‐duration contract residents satisfying the demand for workers of all types. Census data from 2017 indicate a total population of 2.6 million of which native Qatari make up only around 10% [2]. Large numbers of non‐Qatari residents come from the Indian sub‐continent, the Philippines or other Arabic countries, including some from other Gulf Cooperation Countries. A considerable number of these non‐Qatari residents are employer‐sponsored workers in construction (males) or domestic work (females). Non‐Qatari residents contribute to the population count, but not to family units or child raising, although this is changing over time. As a result, native Qatari children make up around a quarter of the child population with the next biggest groups being Arabic and Indian sub‐continent [2].

The status of Qatari nationals and the size and diversity of the non‐Qatari population is challenging for health planning. Much of Qatar's strategic focus is on Qatari nationals, as the long‐term Indigenous residents of the country. Most health spending and planning has been directed as a priority to Qatari nationals [3]. However, increasing numbers of non‐Qatari nationals are long‐term contract residents or their dependents in families and are increasingly in scope for health spending and future planning [2].

Qatari faces numerous health issues including oral diseases. National studies of children in Qatar have documented high levels of childhood primary dentition caries prevalence and experience. The National Oral Health Survey 2011 (NOHS 2011) estimated the prevalence and experience of primary dentition caries for the 6‐year‐old child population at 72% and the sum of the decayed, missing due to caries (extracted) or filled primary teeth (dmft) was 4.2 teeth [4]. The Qatar Child Oral Health Survey 2017 (QCOHS 2017) focused on the oral health of 4–8‐year‐old children [5]. Nearly three‐quarters (69.3%) of children 4–8‐year‐old had experienced caries in their primary teeth and on average they have 3.8 primary teeth with caries experience.

These studies also described bivariate comparisons of nationality categorised as Qatari or non‐Qatari against oral health measures. The prevalence of primary dentition caries was 83.5% among Qatari 4–8‐year‐old children compared with 65.2% among non‐Qatari children. Caries experience was also higher among Qatari children with a dmft of 4.7 compared with 3.5 primary dentition teeth among non‐Qatari children [5]. Several aspects of this situation are important. First, unlike many countries Qatari nationals are only a small minority of the population. Non‐Qataris are the vast majority within the population, with several subgroups several fold larger than that of the Qatari nationals by country of origin. In many countries, those of an immigrant nationality generally have more health problems, including oral health problems. Successive waves of children of immigrants into Australia have had poorer oral health [6]. This does not seem to be the case in Qatar at least at the level of the broad categorization of non‐Qatari.

The size of the non‐Qatari child population and its likely diversity of nationalities creates an interesting case study for the role of nationality in oral health. Non‐Qatari children vary in geographic areas from which they hail [2] and dominant shared cultural and behavioural backgrounds that may impact on and shape health [1]. Therefore, there were two study objectives. First, to understand the caries prevalence and experience differences within the child population classified into four nationality groups, and secondly to explore the factors behind differences.

## 2 Methods

This study is reported according to STROBE (Strengthening the Reporting of Observational Studies in Epidemiology) guidelines [7].

### 2.1 Participating Children

The target population was children resident in Qatar aged 4–8 years. The target sample size was determined to detect an effect size of 0.3 with a statistical power of 80%, a design effect of 1.5 and alpha of 0.05 among 4–5‐year‐olds combined and 6–8‐year‐olds combined based on the data on 6‐year‐old children in NOHS 2011 [4].

A stratified two‐stage sample design was implemented [5]. In the first stage of selection, a sampling frame of schools was compiled from the Supreme Council on Education that listed community‐related to embassy, independent/Arabic private Qatar curriculum, and international curriculum kindergartens/schools located within three geographic regions of Qatar (Northern, Central and Western). Kindergartens and primary schools were then selected using systematic sampling with probability of selection proportional to enrolment size. Children were selected based on their date of birth and age at a reference or chosen date at the time of their selection in the early part of 2017. In the second stage of selection, children were then selected in each year of age using systematic sampling with equal probability. Assuming a response rate of 60%, some 15 children from each age of 4 and 5 years old from 20 kindergartens and some 15 children from each age of 6–8 years old were to be initially selected from each of 40 schools.

The study was conducted in accordance with relevant institutional policies and protocols for studies on human subjects. Ethics approval was obtained from the Institutional Review Board at Primary Health Corporation (PHCC/IEC/16/12/033). The selected kindergartens/schools were approached for participation and to gain local cooperation. Positive informed consent from parents for their child to participate was obtained as the lead into a parental questionnaire.

### 2.2 Data Collection

A parental questionnaire was used to collect both a socio‐demographic and socio‐economic measures and proxy‐reported child dental preventive and risk behaviours. These data items were tailored for use in Qatar from the NCOHS 2012–14 parental questionnaire used in Australia [6], then translated into Arabic and a dual‐language Arabic and English version used in Qatar. The final questionnaire underwent a pilot test with parents in Qatar. Parents were able to complete the questionnaire and provided useful feedback on some minor rewording and the additional of pictorial descriptions of quantity sizes for the dietary information on sugary foods and beverages.

Standardised oral epidemiological examinations were conducted by a team of 10 trained examiners. Examinations took place in kindergartens/schools using portable dental equipment. Information from the oral epidemiological examination was captured directly onto laptop computers using a customised MS Access database.

The examinations involved visual assessment only, using a mirror, blunt probe, and compressed air. Cavitated carious lesions were observed and recorded, along with restorations at the surface‐level and teeth missing due to caries. The examination protocol was based on that of the National Child Oral Health Survey of Australia (NCOHS) 2012–14 [6]. Surface‐level observations have been aggregated to the tooth‐level for analysis. The examinations did not involve X‐rays and no dental treatment was provided.

Training and calibration of the examination team took place prior to the commencement of the fieldwork. The examiners were tested for compliance with the examination protocol during training and later for reliability during the fieldwork. A total of 37 children were re‐examined to test for reliability during the fieldwork against two local ‘gold’ examiners. The intraclass correlation coefficient (ICC) for caries experience in re‐test against the local gold examiners' scores was 0.98 for dmfs for 4–8‐year‐olds, indicating good to excellent reliability.

Questionnaire data were prepared following detailed coding and data entry instructions. Oral epidemiological data across multiple laptop computers were combined into one data set. A linked data set was prepared. Records for both the parental questionnaire and oral examination were required for a child to be regarded as a full participant. The consent procedure was embedded in the parental questionnaire which was first obtained. Only few children absent from kindergartens or schools at each attempt for an oral examination did not have both records. The rate of consent for participation varied across clusters with an overall rate of 48.1%.

### 2.3 Data Weighting

Participation rates varied by kindergarten, school and age and therefore an initial weight was derived to adjust for these differential rates. The weight was defined as the number of children selected of a specific age divided by the number of children who fully participated (parental questionnaire and oral examination) of that age. The initial weights were then adjusted to ensure that the weighted sex by age sample distribution reflected the 2015 Qatar Census distribution for children aged 4–8 years [8]. Data were analysed using statistical procedures that adjust for complex sampling and varying response rates. Hence, estimates representative of Qatar's child population aged 4–8 years have been produced.

### 2.4 Outcome Variables

The dental examination included primary dentition caries assessments. Derived variables were calculated. In this study, disease experience was expressed as the mean number of decayed (d), missing due to dental caries (m) and filled (f) due to dental caries which allowed us to estimate dmft for primary dentition and the prevalence of caries in the primary dentition, dmft > 0.

### 2.5 Indicators

The set of socio‐demographic indicators included age and sex. Four nationalities were identified (Qatari and N‐Q Arabic, N‐Q Indian sub‐continent and N‐Q other [mostly European]). Allocation was based on one or more parents' place of birth, with a hierarchy that moved from Qatari to Arabic, to Indian, to other. Kindergarten/School type (Community‐related to Embassy, Independent/Arabic, International) and the highest level of Parental education (School/Diploma, University) were also coded.

The set of behavioural variables covered four areas. Toothbrushing behaviour was captured around brushing commencing before age 3 years and frequency of brushing with toothpaste (less than once a day, once a day, twice a day or more than twice a day). The type of toothpaste was also captured as standard fluoride toothpaste, children's toothpaste, non‐fluoride toothpaste or do not know/not sure. Exposure to sugary foods and sugar‐sweetened beverages exposure was categorised into approximate tertiles (low, intermediate, high). The cut points applied were 8 and 13 standard serve counts of sugary food and/or sugar‐sweetened beverages. Standard serve sizes were defined in writing and each were illustrated pictorially in the questionnaire. Dental visiting was captured by whether a visit for a check‐up was made in the last 12 months.

Usual drinking water was identified as bottled water or tap water. Almost all tap water is desalinated water with no fluoride. The drinking of bottled water was based on brands listed as usually drunk which were categorised by the fluoride level tested in 2016. A total of 30 brands of bottled water were identified in fluoride assay results and parental questionnaire responses. However, these were collapsed to 18 brands when different spelling or abbreviations of brands were matched. For one brand no fluoride assay could be identified. Two‐thirds of the brands, 12, of the bottled water brands were categorised as no fluoride bottled water (No F). This included two of the most prevalent brands drunk on a usual day, Rayyan and Al Safa. Of the remaining six low fluoride bottled waters (Low F) half had fluoride levels < 0.3 mg F/L with only three brands (Al Manhal, Nestle, Marwa) tested on multiple occasions to have > 0.3 mg F/L. Al Manhal and Nestle were among the more prevalent brands drunk on a usual day.

Missingness was moderately high on many variables, including nationality which had 16% of children with missing data. The exception was school type which was known at selection. Missing responses, along with Do not know, were excluded.

### 2.6 Analysis

SAS callable procedures from SUDAAN software release 11.0.3 were used. The SUDAAN procedures used weights to generate population estimates that adjusted for the complex sample design used in this research. Specially designed macros were already developed and were modified to generate statistical estimates and 95% confidence intervals of key variables.

The associations of nationality classified into four groups with putative socio‐demographic and behavioural indicators were initially examined. Then a bivariate model was run to explore the association between prevalence and experience of dental caries in primary teeth and the four nationality groups to satisfy the first objective. The analysis included unadjusted prevalence ratios and their 95% confidence intervals (95% CI) for the prevalence of caries and unadjusted mean ratios (MRs) and their 95% CI for caries experience, dmft. Missing nationality was examined both for the outcomes (% children with caries and mean dmft). The two outcomes were compared between those with and without nationality missing. Initially a complete‐case analysis was conducted. Then, multiple imputation was conducted using SAS (PROC MI), imputing 10 datasets (*m* = 10). SUDAAN handled the imputed datasets by analysing each imputed dataset separately and then combing the estimates to produce summary results across all imputed datasets. The bivariate model was rerun with missing data imputed for nationality.

Multivariable models were generated for each of the caries prevalence and experience building from nationality alone, through socio‐demographic indicators, then behavioural indicators and finally a full model.

Adjusted prevalence ratios and their 95% confidence intervals (95% CI) for the prevalence of caries and adjusted mean ratios (MRs) and their 95% CI for caries experience, dmft, were calculated. The focus was on the significance of the association of caries with nationality across these models and to a lesser extent the putative indicators which were significantly associated with caries prevalence and caries experience. This informed about the second objective. Analysis was conducted using multivariable log‐Poisson regression with robust standard error estimation using PROC LOGLINK. Initially a complete‐case analysis was conducted. Then, multiple imputation was conducted using SAS (PROC MI), imputing 10 datasets (*m* = 10). SUDAAN handled the imputed datasets by analysing each imputed dataset separately and then combing the estimates to produce summary results across all imputed datasets. The multivariate models were rerun with missing data imputed for nationality.

## 3 Results

Table 1 presents the weighted data showing that Qatari nationality children made up just over a quarter of children (26.3%), while non‐Qatari (N‐Q) Arabic nationality children were the largest group (44.2%). There were smaller percentages of N‐Q Indian sub‐continent and N‐Q Other (mostly European) nationality children.

**TABLE 1:** Socio‐demographic indicators (age, sex, school type, parent highest level of education) by nationality of parents.

Indicators + categories	Distribution	Qatari	Non‐Qatari–Arabic	Non‐Qatari–Indian sub‐cont	Non‐Qatari –other (mostly European)
All children	100.00	26.3% (18.9–35.3)	44.2 (35.2–53.6)	16.4 (9.2–27.5)	13.1 (7.3–22.5)
Age					
4/5 years	40.9 (27.8–55.3)	43.3 (24.4–64.4)	38.7 (23.0–57.2)	48.8 (21.0–77.4)	46.7 (19.1–76.5)
6/7/8 years	59.1 (44.7–72.2)	56.7 (35.6–75.6)	61.3 (42.8–77.0)	51.2 (22.6–79.0)	53.3 (23.5–80.9)
Sex					
Male	51.1 (43.2–58.9)	45.6 (29.3–63.0)	52.6 (44.5–60.5)	53.3 (44.0–62.4)	49.2 (38.7–59.8)
Female	48.9 (41.1–56.8)	54.4 (37.0–70.7)	47.4 (39.5–55.5)	46.7 (37.6–56.0)	50.8 (40.2–61.3)
School type					
Communities related to embassy	7.9 (3.3–17.7)	0.1 (0.0–1.0)	8.3 (2.6–23.3)	1.6 (0.2–12.1)	30.2 (8.3–67.6)
Independent + national curriculum	30.2 (19.2–44.1)	60.7 (41.0–77.3)	25.2 (13.3–42.6)	2.3 (0.7–7.2)	1.1 (0.2–5.0)
International curriculum	61.8 (47.9–74.0)	39.2 (22.6–58.8)	66.5 (49.0–80.4)	96.1 (87.8–98.8)	68.7 (32.1–91.0)
Parent highest level of education					
School/Diploma	26.9 (21.2–33.6)	46.6 (37.2–56.2)	22.8 (17.4–29.2)	12.6 (6.3–23.5)	22.8 (7.3–52.5)
University	73.1 (66.4–78.8)	53.4 (43.8–62.8)	77.2 (70.8–82.6)	87.4 (76.5–93.7)	77.2 (47.5–92.7)

*Note:* Age at time of children's selection in the sample. Missing: Excluded.

Also shown in Table 1, nationality was associated with kindergarten/school type with more Qatari children attending Independent and national curriculum, N‐Q Arabic and N‐Q Other attending International and N‐Q Indian almost exclusively attending International curriculum schools. Nearly three‐quarters of parents had a university‐level education. Many more N‐Q children's parents had a university education.

The distribution of the children across the categories of behavioural variables and their association with nationality are presented in Table 2. Just under 60% of children had not commenced toothbrushing by 3 years old. More Qatari and N‐Q Arabic children were later starters to toothbrushing, and fewer Qatari, N‐Q Arabic and N‐Q Indian children brushed 2+ times per day than N‐Q Other children. Qatari children had the highest percentage in the high sugar exposure group, significantly higher than the N‐Q Other group of children. Fewer Qatari, N‐Q Arabic and especially N‐Q Indian children had made a check‐up visit in the last 12 months than N‐Q Other children. Most children usually drank bottled water, evenly split between those bottled waters with no or negligible fluoride (no F bottled water) and those with a low level of fluoride (low F bottled water). Only a small percentage of children were identified as usually drinking tap water. Most Qatari children (91.2%) drank bottled water with no or negligible fluoride, contrasting strongly with the other nationality groups.

**TABLE 2:** Behavioural indicators (toothbrushing, dietary sugars, dental visiting, drinking fluoridated bottled water) by nationality of parents.

Indicators + categories	Distribution %	Qatari	Non‐Qatari Arabic	Non‐Qatari Indian sub‐cont	Non‐Qatari other (mostly European)
Age of toothbrushing commencement					
Less than 3 years	40.8 (35.4–46.3)	35.6 (27.0–45.3)	35.5 (30.7–40.7)	54.7 (45.7–63.5)	73.7 (64.3–81.4)
3+ years	59.2 (53.7–64.6)	64.4 (54.7–73.0)	64.5 (59.3–69.3)	45.3 (36.5–54.3)	26.3 (18.6–35.7)
Frequency of toothbrushing					
< 1 per day	7.4 (5.0–10.9)	10.1 (4.5–21.5)	10.1 (7.0–14.3)	0.9 (0.3–2.7)	
Once per day	36.9 (32.1–41.9)	33.1 (24.7–42.8)	44.4 (38.6–50.3)	44.1 (33.3–55.6)	16.7 (9.4–27.8)
2+	55.7 (50.0–61.3)	56.8 (46.3–66.6)	45.5 (39.5–51.7)	54.9 (43.6–65.8)	83.3 (72.2–90.6)
Sugar exposures					
Low	35.1 (32.0–38.3)	22.7 (16.7–30.0)	28.3 (23.4–33.8)	40.1 (32.2–48.5)	38.1 (27.9–49.5)
Intermediate	32.3 (28.7–36.1)	33.5 (25.8–42.1)	35.5 (30.2–41.2)	34.5 (24.6–45.9)	37.3 (26.8–49.1)
High	32.6 (28.9–36.5)	43.9 (35.3–52.8)	36.2 (29.8–43.0)	25.5 (16.4–37.2)	24.6 (16.5–35.0)
Dental visiting the last 12 months					
Check‐up	15.2 (11.5–19.8)	12.0 (7.8–18.0)	17.5 (13.0–23.1)	6.4 (3.1–12.7)	36.5 (27.6–46.3)
Other	84.8 (80.2–88.5)	88.0 (82.0–92.2)	82.5 (76.9–87.0)	93.6 (87.3–96.9)	63.5 (53.7–72.4)
Drinking water					
No F bottled water	50.3 (43.6–57.0)	87.2 (81.2–91.6	39.7 (34.7–44.9)	22.7 (16.1–31.1)	44.2 (34.5–54.3)
Low F bottled water	45.4 (39.0–52.0)	8.4 (4.9–14.0)	55.7 (49.8–61.5)	69.4 (62.5–75.6)	55.6 (45.5–65.2)
Tap water	4.2 (2.9–6.1)	4.3 (1.9–9.8)	4.6 (2.5–8.3)	7.8 (4.2–14.1)	0.3 (0.0–2.3)

*Note:* Missing: Excluded.

Table 3 presents the bivariate associations between childhood caries outcomes and nationality. Overall, the prevalence of caries was 69.3% (95% CI 63.4, 74.5%) and the mean caries experience was 3.8 teeth (95% CI 3.3, 4.2). Caries prevalence was lower among N‐Q children with the PRs being 32% and 35% lower for N‐Q Indian and N‐Q Other children. Caries experience was significantly lower among N‐Q children than the reference Qatari nationality children with the MRs being 42% and 39% lower for N‐Q Indian and N‐Q Other children.

**TABLE 3:** Primary caries in Qatar by nationality—bivariate association expressed by unadjusted prevalence ratio and means ratio.

All children 4–8 years				
Indicator + categories	% children with caries (95% CI)	PR (95% CI)	Mean dmft (95% CI)	MR (95% CI)
All children	69.3 (63.4, 74.5)		3.8 (3.3, 4.2)	
Nationality				
Qatari (Ref)	83.5 (76.7, 88.6)	Ref.	4.7 (4.3, 5.2)	Ref.
Arabic	68.9 (61.1, 75.7)	0.82 (0.74, 0.92)	3.7 (3.0, 4.3)	0.78 (0.65, 0.94)
Indian sub‐continent	57.0 (46.8, 66.7)	0.68 (0.56, 0.83)	2.7 (2.2, 3.3)	0.58 (0.46, 0.72)
Other	54.2 (41.1, 66.8)	0.65 (0.51, 0.83)	2.9 (1.8, 4.0)	0.61 (0.42, 0.88)

*Note:* Missing: excluded.

Abbreviations: MR, means ratio; PR, prevalence ratio.

The impact of missing data for nationality with these outcomes was examined. There was no difference in prevalence of caries in the primary dentition or mean dmft between those with nationality identified and those for which it was missing. Prevalence of caries in the primary dentition was 69.3% (95%CI; 63.4%–74.5%) for those with nationality versus 71.6% (095%CI; 59.6%–81.1%) for those with missing data on nationality.

The association between nationality and the outcomes was also examined after missing nationality was imputed. No association of the two outcomes with nationality changed in significance and actual variations in the PRs and MRs were minor. These findings are presented in Appendix Table A1.

Table 4 presents the unadjusted and then adjusted associations of the caries outcomes with nationality in the presence of socio‐demographic and behavioural variables from the complete‐case analysis. The PRs for the association of caries prevalence with nationality and MRs for the association of caries experience with nationality were no longer significant for the N‐Q nationalities compared with Qatari children. The association of caries with nationality was greatly attenuated in the presence of the socio‐demographic and behavioural variables. The variables that were significantly associated with caries prevalence and/or experience were age, parental education, toothbrushing frequency, sugar exposure and check‐up visiting in the last 12 months.

**TABLE 4:** Primary caries prevalence and experience by nationality, socio‐demographic and behavioural variables—bivariate and multivariable models.

Variable	Categories	Bivariate model of prevalence unadjusted PR (95% CI)	Multivariable model of prevalence adjusted PR (95% CI)	Bivariate model of experience unadjusted MR (95% CI)	Multivariable model of experience adjusted MR (95% CI)
Nationality	Qatari (ref)	Ref.	Ref.	Ref.	Ref.
	Arabic	0.82 (0.74, 0.92)	0.89 (0.78, 1.01)	0.78 (0.65, 0.94)	0.92 (0.73, 1.16)
	Indian sub‐continent	0.68 (0.56, 0.83)	0.80 (0.61, 1.06)	0.58 (0.46, 0.72)	0.79 (0.57, 1.11)
	Other	0.65 (0.51, 0.83)	0.80 (0.61, 1.05)	0.61 (0.42, 0.88)	0.94 (0.61, 1.44)
Age	4/5 years		0.80 (0.70, 0.91)		0.90 (0.73, 1.12)
	6/7/8 years		Ref.		Ref.
Sex	Male		Ref.		Ref.
	Female		1.08 (0.95, 1.22)		0.99 (0.82, 1.18)
School type	Communities related to embassy		0.85 (0.64, 1.12)		0.86 (0.58, 1.28)
	Independent + National curriculum		Ref.		Ref.
	International curriculum		0.90 (0.81, 1.01)		0.93 (0.75, 1.14)
Parent highest level of education	School/Diploma		Ref.		Ref.
	University		0.87 (0.78, 0.97)		0.74 (0.61, 0.89)
Toothbrushing—Age of commencement	Less than 3		0.96 (0.87, 1.06)		0.89 (0.72, 1.09)
	3+		Ref		Ref
Toothbrushing—Frequency	< 1 per day		Ref		Ref
	Once per day		0.96 (0.79, 1.15)		0.89 (0.63, 1.25)
	2+		0.95 (0.80, 1.12)		0.73 (0.54, 0.97)
Sugar exposures	Low		0.90 (0.78, 1.04)		0.81 (0.66, 0.98)
	Intermediate		0.89 (0.80, 1.00)		0.88 (0.72, 1.07)
	High		Ref		Ref
Dental visiting—check‐up in last 12 months	Check‐up		0.71 (0.56, 0.90)		0.65 (0.45, 0.95)
	Other		Ref		Ref
Drinking water	No F bottled water		1.04 (0.92, 1.17)		0.89 (0.69, 1.15)
	Low F bottled water		Ref		Ref
	Tap water		1.01 (0.67, 1.52)		0.97 (0.59, 1.59)

*Note:* Age at time of children's selection in the sample. Missing: Excluded.

Abbreviations: MR, means ratio; PR, prevalence ratio.

The multivariate models of the caries outcomes with nationality in the presence of socio‐demographic and behavioural variables from the analysis using multiple imputation produced little change in estimates for the unadjusted and adjusted associations. The finding using multiple imputation are presented in Appendix Table A2.

## 4 Discussion

The key findings were that primary dentition caries in the Qatari population aged 4–8 years varied by nationality with non‐Qatari Arabic, Indian sub‐continent and other (mostly European) children having a lower prevalence of caries (dmft > 0) and caries experience (dmft score) than Qatari nationality children. Indian sub‐continent and other (mostly European) children had similar caries prevalence and experience. Whilst non‐Qatari Arabic children had slightly higher prevalence and experience of caries, Qatari children still had significantly higher prevalence and experience than all three other nationality groups However, the analysis of caries prevalence and experience in the presence of socio‐demographic and behavioural variables showed the association of caries with nationality was attenuated. Qatari children had higher prevalence and experience of caries, but the differences were non‐significant. The residual non‐significant differences may have been due to omitted indicators. Household income was omitted from the socio‐demographic indicators because of its high association with nationality. Qatari children had the systematic privilege of higher income than most non‐Qatari children. Some indicators may also have been less precise than desirable. For instance, the exposure to sugars was captured with measures developed for Australia with only minor accommodation of the eating of dates.

A strength of this study was the probability‐based population sampling strategy and the ability to weight data to represent the child population in Qatar and the identification of several non‐Qatari nationality groups. While the participation rate in both aspects of data collection was disappointing, adequate numbers of children from Qatari, N‐Q Arabic and N‐Q Indian nationalities were obtained. The number of N‐Q Other (mostly European) was lower, increasing the confidence interval around estimates for this group. Children were clustered within schools. Within schools, children were selected from lists of all children sorted by age at a reference date. Hence, there was clustering by school and age. The sample weighting process using schools as clusters took such a clustering effect into account. Hence, population‐representative estimates were obtained as required for our objectives. Multi‐level analysis can also adjust for clustering, but it was not considered necessary in the analysis as there was no need to model variance within schools and the objective was not to estimate effects of school‐level factors.

Another strength was the extensive parental questionnaire, and the detailed oral examination conducted by trained examiners under standard conditions. Missing data were an issue with the parental questionnaire. The level of missingness for nationality was reasonably high and missingness was associated with the four nationality groups. Nationality was missing either due to a lack of answers on the questionnaire or coding difficulties. The dual‐language questionnaire may not have been readily completed by all nationality groups. Missing nationality was not associated with key socio‐demographic indicators such as age, sex or school type. It was associated with parent highest level of education, with higher levels of missing nationality for those with school or diploma education than university. Comparison of the outcomes between those with and without the nationality variable and after multiple imputation showed that missingness may have introduced minimal bias. This was considered minor and did not change the direction of the key findings.

This study focused on children resident in Qatar aged 4–8 years. The 4‐ and 5‐year‐old age group was selected as those children are in kindergartens, while the older children, 6–8‐year‐olds, were at school. The age range excluded children less than 4 years old and therefore does not lend itself to estimating severe early childhood caries (S‐ECC). However, the 4 and 5 years old estimates are comparable to early childhood caries (ECC) with a threshold of cavitation for decay. For children older than 6 years it is possible that caries may affect both the primary and permanent teeth, therefore looking at dmft may underestimate the overall prevalence of dental caries.

In the Gulf Cooperation Council countries, caries prevalence and experience are high, and numerous studies have found caries to be higher in native or Indigenous children. Being native or indigenous has been described as a risk indicator for dental caries [9]. Nationality could operate through factors at the family level and broader social, cultural and physical factors at a community level. In this study nationality was largely a proxy for socio‐demographic and behavioural differences between population groups that impact on and shape oral health [10].

Nationality was important in identifying the social context like the type of schooling and educational attainment in the home through to behaviours practised including aspects of toothbrushing behaviour with fluoridated toothpaste, exposure to dietary sugars, and dental check‐up visiting. Aspects of the social context are slow to change, like the educational attainment of parents, but others like the school environment can be the targets of campaigns. The very substantial differences between the nationalities in toothbrushing behaviour, exposures to sugars, making a dental visit for a check‐up and even drinking water show the importance of nationality to health behaviours. The findings give direction to effects to modify behaviour. Many studies within the region and internationally have highlighted the association between nationality and oral health status as well as oral health‐related quality of life [11, 12, 13].

The main preventive dental behaviour was toothbrushing with the late age at which toothbrushing with toothpaste commenced and the relatively low percentage reportedly brushing 2+ times a day among all nationalities except N‐Q Other [14]. There was also a low awareness of the need for early preventively oriented check‐up dental visiting among Qatari, N‐Q Arabic and N‐Q Indian groups [15].

A key risk behaviour associated with nationality was exposure to sugary foods and sugar‐sweetened beverages. Sugar intake is strongly related to caries outcomes [16]. The exposure to sugary foods and sugar‐sweetened beverages was high among Qatari children with 44% having 13+ serves (high sugar exposure) on a usual day. Interventions aimed at altering sugar intake should not only consider nationality but also be informed by a finer grain level of analysis of the available data on specific foods and beverages which are frequently consumed. Further information on dietary sugar consumption would be especially helpful if it is thought dietary patterns are changing [9].

The parental questionnaire included a nested study to extend the information available on drinking water consumption by children on a usual day. The brands of bottled water usually drunk by children were identified and matched with information on the fluoride content. While these data from 2015 might seem dated, they were relevant in that they preceded QCOHS 2017. There was considerable variation between nationalities in whether they usually drank bottled water with no fluoride. A much higher percentage of Qatari children drank bottled water with no fluoride than the three N‐Q nationality children. There is an opportunity to increase children's exposure to drinking water with even low levels of fluoride as fluoridated ‘packaged’ water has been deemed equivalent to fluoridated tap water in its caries preventive potential [17]. However, in the presence of all indicators in the multivariable models, the drinking water indicator was not significantly associated with caries outcomes.

Check‐up visiting was low for all nationalities except N‐Q Other. There is a need to promote asymptomatic visiting so that clinical preventive measures can be provided. Periodic check‐up visits also provide an opportunity for early diagnosis and prompt treatment of caries.

On a national‐level, healthcare leaders are endeavouring to ensure a top‐tier healthcare system as part of the country's 2030 vision focusing on integration, quality care and service delivery [18]. Qatar's healthcare system includes various dental service providers (public, semi‐public, and private) providing a diverse range of dental services. Most of which are delivered either free of charge or subsidised by public healthcare providers. Moreover, the Ministry of Public Health started a phased implementation of the National Health Insurance [19].

These considerations fit well with the directions put forward in the National Oral Health Committee Road Map for Qatar in 2016 [20]. The Road Map identified the need for population‐wide exposure to fluorides (including through regulating the fluoride content of bottled water and promoting the appropriate pattern of toothbrushing), oral health promotion through anticipatory guidance for mothers with infants, well‐baby checks, and a settings approach in kindergartens and school for oral disease prevention and oral health promotion for young children [21]. Outreach through kindergarten/school‐based screening and provision of non‐invasive procedures need to be followed up by referral to clinics for more definitive treatment in a primary care setting [21].

## 5 Conclusion

Nationality is an important indicator for primary dentition caries in children in Qatar. However, nationality appears to operate not through a cultural context, but through sociodemographic differences and behavioural practices related to oral health. These differences indicate the pathways to improving primary dentition caries prevalence and experience of children in Qatar.

## Author Contributions

**Andrew John Spencer:** conceptualization, data capture, data curation, writing – original draft. **Asmaa Othman AlKhtib:** principal investigator, conceptualization, data capture, review and editing. **Mohamed Sultan Al Darwish:** co‐investigator, conceptualization, data capture, review and editing. **Hasaan Gassim Saad Mohame:** data capture and statistical data management, review and editing. **Tintu Mathew:** data capture and statistical data management, review and editing. **Ghanim Ali Al Mannai:** support for the research team, review and editing and approval. **Mariam Abdulmalik:** senior responsible officer of the research project, review, editing and approval. **Mohammed Al Thani:** custodian of the research project, review, editing and approving. **Johann de Vries:** conceptualization, review and editing. **Loc Giang Do:** conceptualization, data capture, data curation, formal analysis, writing – review and editing. **Sergio Chrisopoulos:** data capture, data curation, formal analysis, writing – original draft.

## Conflicts of Interest

The authors declare no conflicts of interest.

## Supporting information


Appendix S1.

## Data Availability

The data that support the findings of this study are available on request from the corresponding author. The data are not publicly available due to privacy or ethical restrictions.
