# Supplementary material for: The Role of Nationality in Childhood Caries in Qatar
Source: Community Dent Oral Epidemiol. 2024 Oct 30;53(1):106–16. doi: 10.1111/cdoe.13010 (PMC11754148; doi:10.1111/cdoe.13010)
Supplement: Supplementary file 1 — Appendix S1. [file CDOE-53-106-s001.docx]

Appendix Table A1 Primary caries in Qatar by nationality - bivariate association expressed by unadjusted prevalence ratio and means ratio. (based on imputed data (m=10))

|  |  | | **All children 4-8 years** | | | |
| --- | --- | --- | --- | --- | --- | --- |
| **Indicator** | | **Categories** | **% children with  caries (95%CI)** | **PR (95%CI)** | **Mean dmft (95%CI)** | **MR (95%CI)** |
| **All children** | | | 69.3 (63.4,74.5) |  | 3.8 (3.3,4.2) |  |
| **Nationality** | | |  |  |  |  |
|  | Qatari (Ref) | | 83.5 (76.7,88.6) | Ref. | 4.7(4.3,5.2) | Ref. |
|  | Arabic | | 70.9 (63.6,77.3) | 0.85(0.76,0.94) | 3.9(3.3,4.5) | 0.82(0.70,0.97) |
|  | Indian sub-continent | | 58.8 (49.7,67.4) | 0.70(0.59,0.83) | 2.9(2.3,3.5) | 0.62(0.49,0.77) |
|  | Other | | 53.3 (40.1,66.1) | 0.64(0.50,0.82) | 2.9(1.8,4.0) | 0.61(0.42,0.88) |

PR: Prevalence ratio; MR: Means ratio

Appendix Table A2: Primary caries prevalence and experience by nationality, socio-demographic and behavioural variables – bivariate and multivariable models. – using multiply imputed data (m=10)

| **Variable** | **Categories** | **Bivariate model of prevalence unadjusted  PR (95%CI)** | **Multivariable model of prevalence adjusted PR (95%CI)** | **Bivariate model of experience unadjusted  MR (95%CI)** | **Multivariable model of experience adjusted  MR (95%CI)** |
| --- | --- | --- | --- | --- | --- |
|  |  |  |  |  |  |
| **Nationality** |  |  |  |  |  |
|  | Qatari (Ref) | Ref. | Ref. | Ref. | Ref. |
|  | Arabic | 0.85 (0.76,0.42) | 0.91 (0.81,1.03) | 0.82 (0.70,0.97) | 0.96 (0.78,1.19) |
|  | Indian sub-continent | 0.70 (0.59,0.83) | 0.79 (0.65,0.97) | 0.62 (0.49,0.77) | 0.82 (0.61,1.09) |
|  | Other | 0.64 (0.50,0.82) | 0.81 (0.63,1.06) | 0.61 (0.42,0.88) | 0.94 (0.64,1.42) |
| **Age** |  |  |  |  |  |
|  | 4/5 years |  | 0.83 (0.73,0.94) |  | 0.99 (0823,1.20) |
|  | 6/7/8 years |  | Ref. |  | Ref. |
| **Sex** |  |  |  |  |  |
|  | Male |  | Ref. |  | Ref. |
|  | Female |  | 1.06 (0.96,1.19) |  | 1.00 (0.85, 1.17) |
| **School type** |  |  |  |  |  |
|  | Communities Related to Embassy |  | 0.84 (0.65,1.09) |  | 0.78 (0.55,1.11) |
|  | Independent + National curriculum |  | Ref. |  | Ref. |
|  | International curriculum |  | 0.87 (0.79,0.96) |  | 0.79 (0.65,0.96) |
| **Parent highest level of education** | |  |  |  |  |
|  | School/Diploma |  | Ref. |  | Ref. |
|  | University |  | 0.91 (0.81,1.01) |  | 0.79 (0.85,0.97) |
| **Toothbrushing- Age of commencement** | |  |  |  |  |
|  | Less than 3 |  | 0.92 (0.84,1.01) |  | 0.87 (0.74,1.02) |
|  | 3+ |  | Ref |  | Ref |
| **Toothbrushing- Frequency** | |  |  |  |  |
|  | <1 per day |  | Ref |  | Ref |
|  | once per day |  | 0.95 (0.82,1.10) |  | 0.91 (0.66,1.24) |
|  | 2+ |  | 0.94 (0.82,1.08) |  | 0.76 (0.58,0.99) |
| **Sugar exposures** | |  |  |  |  |
|  | Low |  | 0.92 (0.82,1.02) |  | 0.86 (0.72,1.03) |
|  | Intermediate |  | 0.94 (0.84,1.05) |  | 0.95 (0.77,1.16) |
|  | High |  | Ref |  | Ref |
| **Dental visiting – check-up in last 12 months** | |  |  |  |  |
|  | Check-up |  | 0.71 (0.57,0.90) |  | 0.68 (0.49,0.96) |
|  | Other |  | Ref |  | Ref |
| **Drinking water** | |  |  |  |  |
|  | No F Bottled water |  | 1.01 (0.90,1.14) |  | 0.93 (0.75,1.14) |
|  | Low F Bottled water |  | Ref |  | Ref |
|  | Tap water |  | 0.93 (0.70,1.25) |  | 0.92 (0.61,1.39) |

PR: Prevalence ratio; MR: Means ratio

Age at time of children’s selection in the sample.
